# Patient- and Community-Level Characteristics Associated With Respiratory Syncytial Virus Vaccination

**DOI:** 10.1001/jamanetworkopen.2025.2841

**Published:** 2025-04-01

**Authors:** Diya Surie, Katharine A. Yuengling, Basmah Safdar, Adit A. Ginde, Ithan D. Peltan, Samuel M. Brown, Manjusha Gaglani, Shekhar Ghamande, Robert L. Gottlieb, Cristie Columbus, Nicholas M. Mohr, Kevin W. Gibbs, David N. Hager, Mary O'Rourke, Michelle N. Gong, Amira Mohamed, Nicholas J. Johnson, Jay S. Steingrub, Akram Khan, Abhijit Duggal, Jennifer G. Wilson, Nida Qadir, Steven Y. Chang, Christopher Mallow, Laurence W. Busse, Jamie Felzer, Jennie H. Kwon, Matthew C. Exline, Ivana A. Vaughn, Mayur Ramesh, Adam S. Lauring, Emily T. Martin, Jarrod M. Mosier, Estelle S. Harris, Adrienne Baughman, Sydney A. Swan, Cassandra A. Johnson, Paul W. Blair, Nathaniel M. Lewis, Sascha Ellington, Rachel E. Rutkowski, Yuwei Zhu, Wesley H. Self, Fatimah S. Dawood

**Affiliations:** 1 Coronavirus and Other Respiratory Viruses Division, National Center for Immunization and Respiratory Diseases, Centers for Disease Control and Prevention (CDC), Atlanta, Georgia; 2 School of Medicine, Yale University School of Medicine, New Haven, Connecticut; 3 Department of Emergency Medicine, University of Colorado School of Medicine, Aurora; 4 Department of Pulmonary and Critical Care Medicine, Intermountain Medical Center, Murray, Utah; 5 Department of Pulmonary and Critical Care Medicine, University of Utah, Salt Lake City; 6 Center for Research in Vaccines and Infections, Baylor Scott and White Health, Temple, Texas; 7 Center for Research in Vaccines and Infections, Baylor Scott and White Health, Dallas, Texas; 8 College of Medicine, Baylor College of Medicine, Temple, Texas; 9 Burnett School of Medicine, Texas Christian University, Fort Worth; 10 College of Medicine, Texas A&M University, Dallas; 11 University of Iowa, Iowa City; 12 Department of Medicine, Wake Forest School of Medicine, Winston-Salem, North Carolina; 13 Department of Medicine, Johns Hopkins University School of Medicine, Baltimore, Maryland; 14 Department of Emergency Medicine, Hennepin County Medical Center, Minneapolis, Minnesota; 15 Department of Medicine, Montefiore Medical Center, Albert Einstein College of Medicine, Bronx, New York; 16 Department of Emergency Medicine, Division of Pulmonary, Critical Care and Sleep Medicine, University of Washington, Seattle; 17 Department of Medicine, Baystate Medical Center, Springfield, Massachusetts; 18 Department of Medicine, Oregon Health and Science University, Portland; 19 Department of Medicine, Cleveland Clinic, Cleveland, Ohio; 20 Department of Emergency Medicine, Stanford University School of Medicine, Stanford, California; 21 Department of Medicine, University of California, Los Angeles, Los Angeles; 22 Department of Medicine, University of Miami, Miami, Florida; 23 Department of Medicine, Emory University, Atlanta, Georgia; 24 Department of Medicine, Washington University, St Louis, Missouri; 25 Department of Medicine, The Ohio State University, Columbus; 26 Department of Public Health Sciences, Henry Ford Health, Detroit, Michigan; 27 Division of Infectious Diseases, Henry Ford Health, Detroit, Michigan; 28 Department of Internal Medicine, University of Michigan, Ann Arbor; 29 Department of Microbiology and Immunology, University of Michigan, Ann Arbor; 30 School of Public Health, University of Michigan, Ann Arbor; 31 Department of Emergency Medicine, University of Arizona, Tucson; 32 Department of Medicine, University of Utah, Salt Lake City; 33 Department of Emergency Medicine, Vanderbilt University Medical Center, Nashville, Tennessee; 34 Department of Biostatistics, Vanderbilt University Medical Center, Nashville, Tennessee; 35 Vanderbilt Institute for Clinical and Translational Research, Vanderbilt University Medical Center, Nashville, Tennessee; 36 Influenza Division, National Center for Immunization and Respiratory Diseases, CDC, Atlanta, Georgia

## Abstract

**Importance:**

In 2023, the first respiratory syncytial virus (RSV) vaccines were recommended for US adults 60 years or older, but few data are available about which patients were most likely to receive vaccine to inform future RSV vaccine outreach efforts.

**Objective:**

To assess patient- and community-level characteristics associated with RSV vaccine receipt and patient knowledge and attitudes related to RSV disease and RSV vaccines.

**Design, Setting, and Participants:**

During the first season of RSV vaccine use from October 1, 2023, to April 30, 2024, adults 60 years or older hospitalized with RSV-negative acute respiratory illness were enrolled in this cross-sectional study from 26 hospitals in 20 US states. Sociodemographic and clinical data were abstracted from health records, and structured interviews were conducted for knowledge and attitudes about RSV disease and RSV vaccines.

**Exposures:**

Age, sex, race and ethnicity, pulmonary disease, immunocompromised status, long-term care facility residence, medical insurance, social vulnerability index (SVI), and educational level.

**Main Outcomes and Measures:**

The exposures were identified a priori as possible factors associated with RSV vaccine receipt and were entered into a modified Poisson regression model accounting for state clustering, to assess for association with RSV vaccine receipt. Knowledge and attitudes were summarized with frequencies and proportions.

**Results:**

Among 6746 hospitalized adults 60 years or older, median age was 73 (IQR, 66-80) years and 3451 (51.2%) were female. Among the 6599 patients with self-reported race and ethnicity, 699 (10.6%) were Hispanic, 1288 (19.5%) were non-Hispanic Black, 4299 (65.1%) were non-Hispanic White, and 313 (4.7%) were other race or ethnicity. There were 700 RSV-vaccinated (10.4%) and 6046 unvaccinated (89.6%) adults. Among 3219 unvaccinated adults who responded to RSV knowledge questions, 1519 (47.2%) had not heard of RSV or were unsure; 2525 of 3218 (78.5%) were unsure if they were eligible for RSV vaccine or thought they were not. In adjusted analyses, characteristics associated with RSV vaccination were being 75 years or older (adjusted risk ratio [ARR], 1.23; 95% CI, 1.10-1.38, *P* < .001), being male (ARR, 1.15; 95% CI, 1.01-1.30; *P* = .04), and having pulmonary disease (ARR, 1.39; 95% CI, 1.16-1.67; *P* < .001), immunocompromised status (ARR, 1.30; 95% CI, 1.14-1.48; *P* < .001), low (ARR, 1.47; 95% CI, 1.18-1.83, *P* < .001) or moderate (ARR, 1.47; 95% CI, 1.21-1.79; *P* < .001) SVI, and educational level consisting of 4 or more years of college (ARR, 2.91; 95% CI, 2.14-3.96; *P* < .001), at least some college or technical training (ARR, 1.85; 95% CI, 1.35-2.53; *P* < .001), or grade 12 education or General Educational Development (ARR, 1.44; 95% CI, 1.03-2.00; *P* = .03). RSV vaccination was less likely among residents of long-term care facilities, patients with Medicaid coverage, and uninsured patients.

**Conclusions and Relevance:**

In this cross-sectional study of hospitalized adults, knowledge of RSV disease and RSV vaccine eligibility was low. Older adults and those with certain medical conditions were more likely to have received vaccine, suggesting appropriate prioritization, but sociodemographic differences in vaccine uptake occurred.

## Key Points

**Question:**

What patient- and community-level characteristics are associated with respiratory syncytial virus (RSV) vaccination among hospitalized adults 60 years or older during 2023 to 2024?

**Findings:**

In this cross-sectional analysis of 6746 hospitalized adults in 20 states, RSV vaccination was associated with being 75 years or older and having pulmonary disease, immunocompromised status, low or moderate social vulnerability, and at least grade 12 education or General Educational Development. Residents of long-term care facilities and patients with Medicaid or no insurance were less likely to receive RSV vaccine.

**Meaning:**

These findings suggest that RSV vaccination was appropriately prioritized among older adults and those with certain high-risk conditions in 2023 to 2024, but sociodemographic differences in uptake also occurred.

## Teaser

This cross-sectional study assesses patient- and community-level characteristics associated with respiratory syncytial virus vaccination and related patient knowledge and attitudes among adults 60 years or older hospitalized with acute respiratory illness during the 2023-2024 season.

## Introduction

Respiratory syncytial virus (RSV) disease can be as severe as COVID-19 or influenza and has been estimated to cause 60 000 to 160 000 hospitalizations and 6000 to 10 000 deaths each year among adults aged 65 years or older in the US.^1,2^ In June 2023, the first RSV vaccines were recommended by the Centers for Disease Control and Prevention (CDC) for adults 60 years or older.^2^ Although prelicensure trials demonstrated that RSV vaccines were efficacious in preventing symptomatic RSV-associated lower respiratory tract disease with an acceptable safety profile, the trials identified 6 cases of inflammatory neurologic events (eg, Guillain-Barré Syndrome) after RSV vaccination.^2,3,4^ Given safety considerations, CDC cautiously recommended RSV vaccination during 2023 to 2024 using shared clinical decision-making (SCDM) to allow patients and health care professionals to weigh individual risks and benefits of RSV vaccination. Since then, several studies have documented that RSV vaccines effectively reduced the risk of RSV-associated hospitalization among adults 60 years or older.^5,6,7,8^ Additionally, surveys of health care professionals have shown that implementing SCDM recommendations is difficult.^6,9,10^ These findings led CDC to update adult RSV vaccination recommendations in June 2024 to more clearly delineate risk groups by recommending a single dose of RSV vaccine for adults aged 60 to 74 years at increased risk of severe RSV disease and all adults 75 years or older.^6^

Data on vaccination coverage and acceptance of COVID-19 and influenza vaccines document persistently suboptimal uptake with clear sociodemographic differences in uptake, despite universal vaccination recommendations for all persons 6 months or older for both COVID-19 and influenza.^11^ During the 2023-2024 respiratory virus season, national vaccine coverage surveys estimated that 24% of US adults 60 years or older received an RSV vaccine,^12^ but few data describe which patients in this age group were most likely to receive vaccine. Identifying facilitators and barriers to RSV vaccine acceptance and receipt is important to guide outreach and communication about RSV vaccines in the future. Additionally, identifying sociodemographic differences in vaccine receipt can inform focused interventions and efforts to improve access to and information on RSV vaccines. Using data from a multicenter surveillance network in 20 US states, we assessed patient- and community-level characteristics associated with RSV vaccination and patient knowledge and attitudes related to RSV disease and RSV vaccines among adults 60 years or older hospitalized with RSV-negative acute respiratory illness (ARI) during the 2023-2024 season.

## Methods

### Study Design and Participant Eligibility

The Investigating Respiratory Viruses in the Acutely Ill (IVY) Network is a multicenter surveillance network that prospectively enrolls adults hospitalized with ARI at 26 US hospitals in 20 US states. The IVY Network’s primary objective is estimating the effectiveness of COVID-19, influenza, and RSV vaccines as previously described.^5,13,14^ Adults 18 years or older are eligible for enrollment if they are hospitalized with ARI and have had clinical testing for RSV, SARS-CoV-2, or influenza within 10 days of illness onset and 3 days of hospital admission; all patients also have nasal swabs collected and systematically tested for these viruses as part of surveillance. Sites aim to enroll virus-positive and virus-negative patients in a ratio of approximately 1:2. Patients were included in this cross-sectional analysis if they were hospitalized within the IVY Network during October 1, 2023, to April 30, 2024, were 60 years or older, and had negative test results for RSV. Further information is available in eMethods of Supplement 1.

This activity was reviewed by the CDC and each participating institution in the IVY Network, deemed public health surveillance and not research with a waiver of participant informed consent, and was conducted consistent with applicable federal law and CDC policy (45 CFR part 46.102(l)(2); 21 CFR part 56; 42 USC §241(d); 5 USC §552a; 44 USC §3501, et seq). This study is reported following the Strengthening the Reporting of Observational Studies in Epidemiology (STROBE) reporting guideline.

### Data Collection

Demographic and clinical data were obtained through patient interview and abstraction from electronic medical records (EMRs) by trained personnel. Patients’ addresses were converted into geocodes and used to determine the social vulnerability index (SVI) for each patient. The SVI is a community-level measure that is derived from 16 US census variables grouped in 4 domains (socioeconomic status, household composition and disability, racial and ethnic minority status, and housing type and transportation) to provide a composite measure of overall vulnerability.^15^ It was originally designed to identify communities at risk for adverse outcomes during disaster events where higher SVI indicates increased risk; more recently, individuals’ SVI has been shown to be associated with reduced uptake of influenza and COVID-19 vaccines.^16,17,18^ In this analysis, SVI was grouped into tertiles: low (0-0.39), moderate (0.40-0.59), and high (0.60-1.00).

At enrollment, patients completed a standardized interview that collected information about race, ethnicity, employment, educational level, residence type, and health care utilization in the previous year as well as RSV, COVID-19, and influenza vaccination status and knowledge and attitudes about RSV disease and RSV vaccines. Race and ethnicity were categorized as Hispanic or Latino of any race, non-Hispanic Black or African American, non-Hispanic White, and other non-Hispanic race (including American Indian or Alaska Native, Asian, Native Hawaiian or Other Pacific Islander, and self-reported as other). Data on race and ethnicity were collected because of their potential association with RSV vaccine receipt. Questions on knowledge and attitudes about RSV disease and vaccines were adapted from previous influenza studies.^19,20,21^ Knowledge of RSV disease was assessed by asking the question, “Have you heard of RSV?” Knowledge of RSV vaccine eligibility was assessed by asking, “Are you eligible to receive an RSV vaccine?” Attitudes about RSV vaccination were assessed by asking whether the patient had received RSV vaccination and reasons for receipt or nonreceipt.

RSV, COVID-19, and influenza vaccination status were identified from EMRs, state or city registries, and self-report. Final vaccination status was determined by combining data from verified documented sources (EMR and registry data) with plausible self-report (ie, ability to recall date and location of vaccination).^22^ RSV vaccination was defined as receipt of a single dose of RSV vaccine at any time before hospital admission.

### Statistical Analysis

Sociodemographic and clinical characteristics of patients were summarized with frequencies and proportions for the overall population and by RSV vaccination status. χ^2^ Tests were performed to determine statistically significant differences, at a 95% confidence level, between patients with and without RSV vaccination. Rao-Scott adjustments for clustering at the state level were applied to all χ^2^ tests to account for clustering effects.

To assess the association between sociodemographic or clinical characteristics and RSV vaccination status, crude risk ratios (RRs) and adjusted RRs (ARRs) and 95% CIs were estimated using modified Poisson regression models with robust error variance, accounting for within-state clustering. Age, sex, race and ethnicity, presence of pulmonary disease, immunocompromised status, residence in a long-term care facility, medical insurance, SVI, and educational level were identified a priori as potential factors associated with RSV vaccination and were included in the full model as categorical variables. Influenza and COVID-19 vaccination status were considered as potential descending proxies for unmeasured intermediate variables (eg, access to preventive care or vaccine acceptance) in the association between participant characteristics and receipt of RSV vaccine and were therefore not included in multivariable models. Descending proxies have been previously defined as downstream consequences of an unmeasured intermediate variable.^23^ Race and ethnicity were missing for 147 patients (2.2%); long-term care facility status, for 162 (2.4%); and SVI data, for 757 (11.2%). These missing data were excluded from models using listwise deletion. To mitigate the potential bias resulting from missing data about patient educational level for 2138 patients (31.7%), multiple imputation was used by chained equations. Fifty imputed datasets were generated, and separate analyses were conducted for each one, pooling the results according to Rubin rules.^24,25^

Knowledge and attitudes toward RSV disease and vaccination were summarized using frequencies and proportions. Knowledge responses were analyzed among all respondents, by RSV vaccination status, and by patients’ educational level and community SVI among those who self-reported being unvaccinated. χ^2^ Tests with Rao-Scott adjustment for clustering at the state level were used to compare the proportions of participant responses in subgroup analyses. Attitudes were summarized in frequencies and proportions based on reasons for RSV vaccine receipt or nonreceipt. Free-text responses to the “other” category were either reclassified according to existing categories or reported as separate subcategories. Analyses were conducted using SAS, version 9.4 (SAS Institute Inc). Two-sided *P* < .05 indicated statistical significance.

## Results

**Figure 1.**
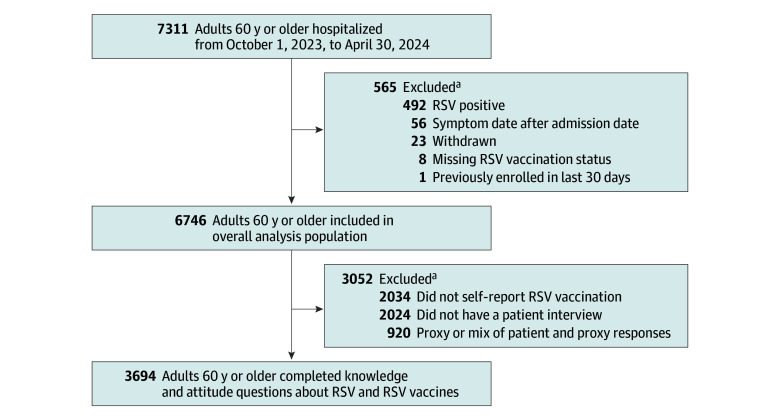
Study Flow Diagram RSV indicates respiratory syncytial virus. ^a^Components of exclusion are not mutually exclusive.

A total of 6746 hospitalized adults 60 years or older were included in this analysis (Figure 1). Median age was 73 (IQR, 66-80) years, 3451 patients (51.2%) were female, and 3295 (48.8%) were male. Among the 6599 patients with self-reported race and ethnicity, 699 (10.6%) were Hispanic, 1288 (19.5%) were non-Hispanic Black, 4299 (65.1%) were non-Hispanic White, and 313 (4.7%) were other race or ethnicity. A total of 6054 patients (89.7%) had at least 1 outpatient visit in the previous year (Table 1 and eTable 1 in Supplement 1). There were 700 RSV-vaccinated (10.4%) and 6046 unvaccinated (89.6%) patients. Of the 700 vaccinated patients, location of RSV vaccination was known for 456 patients (65.1%); the most frequently reported location was a pharmacy or grocery store (338 [74.1%]) followed by a physician’s office (81 [17.8%]) (eFigure 1 in Supplement 1). Compared with unvaccinated patients, RSV-vaccinated patients were more likely to have also received the 2023-2024 COVID-19 vaccine (523 [74.7%] vs 935 [15.5%]; *P* < .001) or influenza vaccine (627 [89.6%] vs 2242 [37.1%]; *P* < .001). Of the 700 RSV-vaccinated patients, 156 (22.3%) received the vaccine on the same day as receiving a COVID-19 vaccine and 56 (8.0%) on the same day as an influenza vaccine.

**Table 1.** Characteristics of Adults Hospitalized With RSV-Negative Acute Respiratory Illness^a^

Characteristic	Participant group, No. (%)			*P* value
	All (N = 6746)	RSV vaccination status		
		Vaccinated (n = 700)	Unvaccinated (n = 6046)	
Age, median (IQR), y	73 (66-80)	75 (69-81)	72 (66-80)	<.001
Age group, y				
60-74	3838 (56.9)	334 (47.7)	3504 (58.0)	<.001
≥75	2908 (43.1)	366 (52.3)	2542 (42.0)	
Sex				
Female	3451 (51.2)	332 (47.4)	3119 (51.6)	.90
Male	3295 (48.8)	368 (52.6)	2927 (48.4)	
Self-reported race and ethnicity				
Hispanic or Latino of any race	699 (10.6)	34 (4.9)	665 (11.2)	<.001
Non-Hispanic Black	1288 (19.5)	61 (8.9)	1227 (20.8)	
Non-Hispanic White	4299 (65.1)	561 (81.7)	3738 (63.2)	
Non-Hispanic other race^b^	313 (4.7)	31 (4.5)	282 (4.8)	
Any outpatient visits in past year	6054 (89.7)	670 (95.7)	5384 (89.1)	<.001
Organ systems with a chronic medical condition^c^				
Cardiovascular disease	5614 (83.2)	571 (81.6)	5043 (83.4)	.22
Pulmonary disease	2954 (43.8)	337 (48.1)	2617 (43.3)	.11
Neurologic disease	1404 (20.8)	126 (18.0)	1278 (21.1)	.01
Endocrine disease	3124 (46.3)	312 (44.6)	2812 (46.5)	.11
Kidney disease	1860 (27.6)	193 (27.6)	1667 (27.6)	.99
Gastrointestinal tract disease	522 (7.7)	48 (6.9)	474 (7.8)	.14
Hematologic disease	1267 (18.8)	143 (20.4)	1124 (18.6)	.43
Immunocompromised status^d^	1577 (23.4)	219 (31.3)	1358 (22.5)	<.001
Long-term care facility resident^e^	726 (10.8)	53 (7.6)	673 (11.1)	.0001
Medical insurance				
Medicare	4824 (71.5)	544 (77.7)	4280 (70.8)	<.001
Medicaid	473 (7.0)	12 (1.7)	461 (7.6)	
Private insurance	1099 (16.3)	117 (16.7)	982 (16.2)	
Other insurance^f^	228 (3.4)	24 (3.4)	204 (3.4)	
Uninsured	122 (1.8)	3 (0.4)	119 (2.0)	
Social vulnerability index, median (IQR)^g^	0.56 (0.28-0.84)	0.39 (0.19-0.62)	0.58 (0.29-0.85)	<.001
Low (0.0-0.39)	2173 (36.3)	327 (51.8)	1846 (34.5)	
Moderate (0.40-0.59)	1064 (17.8)	136 (21.6)	928 (17.3)	
High (0.60-1.00)	2752 (46.0)	168 (26.6)	2584 (48.2)	
Employed^h^	569 (9.8)	60 (9.4)	509 (10.0)	.62
Educational level^i^				
≥4 y of college education	1361 (29.5)	282 (49.3)	1079 (26.7)	<.001
Some college or technical training	1091 (23.7)	129 (22.6)	962 (23.8)	
Grade 12 or GED	1505 (32.7)	131 (22.9)	1374 (34.0)	
Less than high school	651 (14.1)	30 (5.2)	621 (15.4)	
Received 2023-2034 COVID-19 vaccine	1458 (21.6)	523 (74.7)	935 (15.5)	<.001
Received 2023-2024 influenza vaccine	2869 (45.2)	627 (89.6)	2242 (37.1)	<.001

Abbreviations: GED, General Educational Development; RSV, respiratory syncytial virus.

^a^ Data are from 24 hospitals in 19 US states in the Investigating Respiratory Viruses in the Acutely Ill Network, October 1, 2023, through April 30, 2024.

^b^ Includes American Indian or Alaska Native, Asian, Native Hawaiian or Other Pacific Islander, and patients who self-reported their race and ethnicity as other. Data were missing for 147 patients (2.2%).

^c^ Selected based on conditions that may place people at increased risk of severe RSV disease and included the following: cardiovascular disease, pulmonary disease, neurologic disease, endocrine disease, kidney disease, gastrointestinal disease, hematologic disease, and immunosuppression. Details of conditions within each group are provided in eTable 1 in Supplement 1.

^d^ Defined as active solid tumor or hematologic malignant neoplasm (ie, newly diagnosed malignant neoplasm or treatment for a malignant neoplasm within the previous 6 months), solid organ transplant, hematopoietic cell transplant, HIV infection, primary immunodeficiency, use of immunosuppressive medication in the past 30 days, or other conditions that cause moderate or severe immunosuppression.

^e^ Includes patients living in nursing homes, assisted living, rehabilitation hospital, or other subacute or chronic care facility. Data on place of residence before hospitalization were missing for 162 patients (2.4%).

^f^ Includes Tricare, Veterans Affairs, other government insurance, and those who described their insurance status as other.

^g^ The Centers for Disease Control and Prevention and the Agency for Toxic Substances and Disease Registry social vulnerability index (SVI) measures the potential negative effects on communities caused by external stresses on human health by using 16 US census variables to determine social vulnerability for each census tract. Higher SVI values correspond to communities with higher social vulnerability. Data were missing for 757 patients (11.2%).

^h^ Missing for 965 patients (14.3%).

^i^ Missing for 2138 patients (31.7%).

In adjusted analyses, RSV vaccination was associated with being 75 years or older (ARR, 1.23; 95% CI, 1.10-1.38; *P* < .001), being male (ARR, 1.15; 95% CI, 1.01-1.30; *P* = .04), and having pulmonary disease (ARR, 1.39; 95% CI, 1.16-1.67; *P* < .001) and immunocompromised status (ARR, 1.30; 95% CI, 1.14-1.48; *P* < .001). Low (ARR, 1.47; 95% CI, 1.18-1.83, *P* < .001) or moderate (ARR, 1.47; 95% CI, 1.21-1.79; *P* < .001) SVI compared with high SVI and 4 or more years of college (ARR, 2.91; 95% CI, 2.14-3.96; *P* < .001), at least some college or technical training (ARR, 1.85; 95% CI, 1.35-2.53; *P* < .001), or a grade 12 educational level or General Educational Development (ARR, 1.44; 95% CI, 1.03-2.00; *P* = .03) compared with having less than a complete high school education were also associated with vaccine status. Additionally, long-term care facility residents were less likely to have received RSV vaccine (ARR, 0.76; 95% CI, 0.62-0.93; *P* = .01), as were those with Medicaid insurance (ARR, 0.36; 95% CI, 0.20-0.66; *P* < .001) and uninsured patients (ARR, 0.34; 95% CI, 0.12-0.92, *P* = .03) compared with patients with private insurance (Table 2).

**Table 2.** Characteristics Associated with RSV Vaccination Among Adults Hospitalized With RSV-Negative Acute Respiratory Illness^a^

Covariate	Unadjusted RR (95% CI)	*P* value	Adjusted RR (95% CI)^b^	*P* value
Age group, y				
60-74	1 [Reference]	NA	1 [Reference]	NA
≥75	1.40 (1.26-1.56)	<.001	1.23 (1.10-1.38)	<.001
Sex				
Female	1 [Reference]	NA	1 [Reference]	NA
Male	1.20 (1.04-1.39)	.01	1.15 (1.01-1.30)	.04
Self-reported race and ethnicity				
Hispanic or Latino of any race	1 [Reference]	NA	1 [Reference]	NA
Non-Hispanic Black	0.97 (0.52-1.83)	.94	0.79 (0.41-1.51)	.47
Non-Hispanic White	2.45 (1.54-3.89)	<.001	1.35 (0.83-2.20)	.23
Non-Hispanic other race^c^	1.57 (0.92-2.69)	.10	1.02 (0.55-1.89)	.96
Pulmonary disease	1.17(0.96-1.45)	.12	1.39 (1.16-1.67)	<.001
Immunocompromised status^d^	1.44 (1.28-1.62)	<.001	1.30 (1.14-1.48)	<.001
Long-term care facility resident^e^	0.78 (0.64-0.96)	.02	0.76 (0.62-0.93)	.01
Medical insurance^f^				
Medicare	1.14 (1.00-1.31)	.05	1.07 (0.94-1.23)	.27
Medicaid	0.25 (0.13-0.46)	<.001	0.36 (0.20-0.66)	<.001
Private insurance	1 [Reference]	NA	1 [Reference]	NA
Other insurance	1.06 (0.65-1.74)	.82	1.11 (0.72-1.69)	.64
Uninsured	0.25 (0.09-0.67)	.01	0.34 (0.12-0.92)	.03
Social vulnerability index^g^				
Very low or low (0.0-0.39)	2.18 (1.69-2.81)	<.001	1.47 (1.18-1.83)	<.001
Moderate (0.40-0.59)	1.97 (1.60-2.42)	<.001	1.47 (1.21-1.79)	<.001
High or very high (0.60-1.00)	1 [Reference]	NA	1 [Reference]	NA
Educational level				
≥4 y of college education	4.25 (3.05-5.93)	<.001	2.91 (2.14-3.96)	<.001
Some college or technical training	2.40 (1.74-3.32)	<.001	1.85 (1.35-2.53)	<.001
Grade 12 or GED	1.79 (1.31-2.44)	<.001	1.44 (1.03-2.00)	.03
Less than high school	1 [Reference]	NA	1 [Reference]	NA
2023-2024 COVID-19 vaccination	10.05 (8.11-12.45)	<.001	NA^h^	NA
2023-2024 Influenza vaccination	12.82 (9.54-17.23)	<.001	NA^h^	NA

Abbreviations: GED, General Educational Development; NA, not applicable; RR, risk ratio; RSV, respiratory syncytial virus.

^a^ Data are from 24 hospitals in 19 US states in the Investigating Respiratory Viruses in the Acutely Ill Network, October 1, 2023, through April 30, 2024.

^b^ Modified Poisson regression models with robust error variance, accounting for clustering within state, were adjusted for the following variables: age, sex, race and ethnicity, pulmonary disease, immunosuppression, long-term care facility resident, medical insurance, social vulnerability index, and educational level.

^c^ Includes American Indian or Alaska Native, Asian, Native Hawaiian or Other Pacific Islander, and patients who self-reported their race and ethnicity as other.

^d^ Defined as active solid tumor or hematologic malignant neoplasm (ie, newly diagnosed malignant neoplasm or treatment for a malignant neoplasm within the previous 6 months), solid organ transplant, hematopoietic cell transplant, HIV infection, primary immunodeficiency, splenectomy, use of immunosuppressive medication in the past 30 days, or other conditions that cause moderate or severe immunosuppression.

^e^ Includes patients living in nursing homes, assisted living, rehabilitation hospital, or other subacute or chronic care facility.

^f^ Includes Tricare, Veterans Affairs, other government insurance, and those who described their insurance status as other.

^g^ The Centers for Disease Control and Prevention and the Agency for Toxic Substances and Disease Registry social vulnerability index (SVI) measures the potential negative effects on communities caused by external stresses on human health by using 16 US census variables to determine social vulnerability for each census tract. Higher SVI values correspond to communities with higher social vulnerability.

^h^ Receipt of 2023-2024 COVID-19 or influenza vaccine were not included in the models as they were hypothesized to be descending proxies of intermediate variables (eg, access to preventive care, vaccine acceptance) in the association between participant characteristics and receipt of RSV vaccine and were therefore not included in multivariable models. Descending proxies have been previously defined as downstream consequences of an unmeasured intermediate variable.

Among the 6746 hospitalized adults 60 years or older in this analysis with RSV-negative illness, 3694 (54.8%) responded to interview questions about knowledge and attitudes toward RSV disease and vaccination. Those who did not complete interviews (n = 3052) were more likely to be older (median age, 75 [IQR, 68-83] vs 71 [IQR, 65-78] years; *P* < .001), non–English speaking (452 [14.8%] vs 141 [3.8%]; *P* < .001), Hispanic or Latino (433 [14.7%] vs 266 [7.3%]) or other race or ethnicity (183 [6.2%] vs 130 [3.6%]); *P* < .001), and admitted to the intensive care unit (268 of 1153 [23.2%] vs 161 of 1319 [12.2%]; *P* < .001) and to have dementia (339 [11.1%] vs 85 [2.3%]; *P* < .001) compared with patients who responded to interview questions (eTable 2 in Supplement 1).

**Figure 2.**
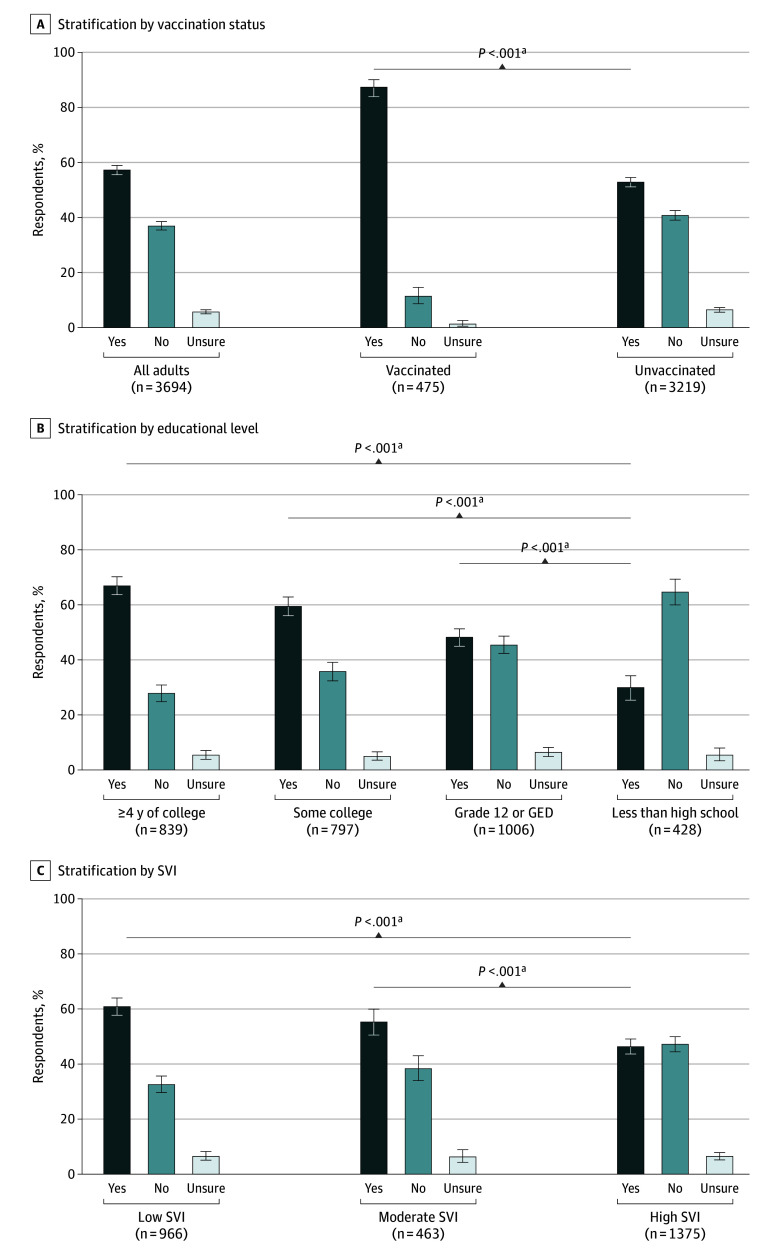
Knowledge of Respiratory Syncytial Virus (RSV) Disease Among Adults 60 Years or Older Hospitalized With RSV-Negative Acute Respiratory Illness A, A total of 3694 adults responded to the question. B, Of 6046 unvaccinated adults, 3070 responded. GED indicates General Educational Development. C, Of 6046 unvaccinated adults, 2804 with information available responded. The social vulnerability index (SVI) is a measure of the potential negative effects on communities caused by external stresses on human health and is based on inputs from 16 US census variables. Higher SVI values correspond to communities with higher social vulnerability; 0 to 0.39 indicated a low SVI; 0.40 to 0.59, moderate SVI; and 0.60 to 1.00, high SVI. Error bars indicate Clopper-Pearson 95% CI. ^a^We used χ^2^ test with Rao-Scott adjustment for within-state clustering to compare the proportions of vaccinated vs unvaccinated patients who reported knowledge of RSV disease. Participants with “no” or “unsure” responses were combined for statistical comparisons.

**Figure 3.**
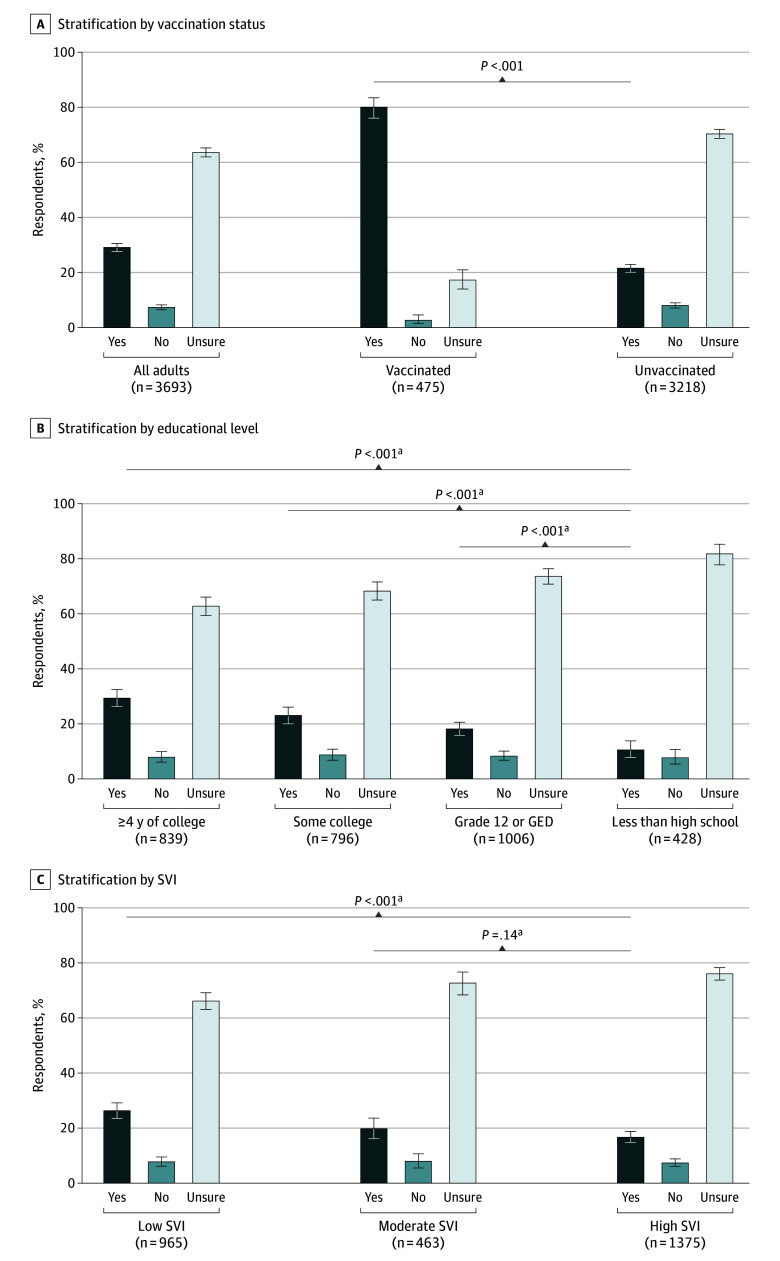
Knowledge of Respiratory Syncytial Virus (RSV) Vaccine Eligibility Among Adults 60 Years or Older Hospitalized With RSV-Negative Acute Respiratory Illness A, A total of 3693 adults responded to the question. B, Of 6046 unvaccinated adults, 3069 responded. GED indicates General Educational Development. C, Of 6046 unvaccinated adults, 2803 with information available responded. The social vulnerability index (SVI) is a measure of the potential negative effects on communities caused by external stresses on human health and is based on inputs from 16 US census variables. Higher SVI values correspond to communities with higher social vulnerability; 0 to 0.39 indicated a low SVI; 0.40 to 0.59, moderate SVI; and 0.60 to 1.00, high SVI. Error bars indicate Clopper-Pearson 95% CI. ^a^We used χ^2^ test with Rao-Scott adjustment for within-state clustering to compare the proportions of vaccinated vs unvaccinated patients who reported knowledge of RSV vaccine eligibility. Participants with “no” or “unsure” responses were combined for statistical comparisons.

Of the 3694 patients who responded to knowledge and attitudes questions about RSV disease and vaccine, 475 (12.9%) were vaccinated and 3219 (87.1%) were unvaccinated. Among 3219 unvaccinated patients, knowledge of RSV was low, with 1519 (47.2%) reporting they had not heard of RSV or were unsure if they had (Figure 2A). RSV knowledge was higher among vaccinated patients than unvaccinated patients (415 of 475 [87.4%] vs 1700 of 3219 [52.8%]; *P* < .001), although 11.4% of patients with documented evidence of RSV vaccination reported that they had not heard of RSV. Among 3219 unvaccinated patients, knowledge of RSV was higher among those with a higher educational level (Figure 2B). For example, 561 of 839 patients (67.0%) with 4 or more years of college education knew about RSV whereas only 128 of 428 (29.9%) with less than a high school education had heard of RSV. Similarly, among 2804 unvaccinated patients with SVI data available, knowledge of RSV was higher among patients residing in communities with low social vulnerability compared with those residing in areas with high social vulnerability (588 of 966 [60.9%] vs 638 of 1375 [46.4%]; *P* < .001) (Figure 2C). When 3693 patients were asked whether they were eligible for RSV vaccines, most were unsure (2349 [63.6%]) or stated they were not eligible (271 [7.3%]); among 3218 unvaccinated patients, proportions were greater (2267 [70.4%] and 258 [8.0%], respectively) (Figure 3A). Similar to knowledge about RSV disease, knowledge of RSV vaccine eligibility was higher among patients with more education and among those residing in communities with low or moderate social vulnerability (Figure 3B-C).

Among the 3694 patients who responded to knowledge and attitude questions, 438 (11.9%) self-reported receipt of RSV vaccine, 2914 (78.9%) reported no receipt of RSV vaccine, and 342 (9.3%) were unsure of their RSV vaccination status (eFigure 2 in Supplement 1). Among 438 patients who self-reported receiving RSV vaccinations, the primary reasons cited for RSV vaccine receipt were that it was important for their health (221 [50.5%]) or that their physician had suggested it (133 [30.4%]). Of 2914 patients who were unvaccinated by self-report, 1216 (41.7%) reported being open to RSV vaccination in the future, and of these, 667 (54.9%) had not received their vaccine yet because they wanted to discuss with a physician first. In addition, 729 of these patients (25.0%) were not open to receiving RSV vaccination in the future, and of these, 180 (24.7%) had concerns about safety of the vaccine, 169 (23.2%) did not know that the vaccine was recommended for them, and 166 (22.8%) did not think that the vaccine was important for their health. Among 107 patients who provided other responses, 32 (29.9%) reported vaccine fatigue, 21 (19.6%) reported that their physician “had advised against RSV vaccination,” and 16 (15.0%) reported concerns related to adverse effects of vaccines.

## Discussion

In this cross-sectional analysis of 6746 adults 60 years and older hospitalized with RSV-negative ARI in 20 US states, only 1 in 10 had received RSV vaccine during the first season of RSV vaccine use. General awareness of RSV disease and RSV vaccine eligibility among the unvaccinated group was low. RSV-vaccinated patients were more likely to be older and have pulmonary disease or immunocompromising conditions, suggesting evidence of appropriate prioritization of vaccine among some groups at increased risk of RSV disease; however, residents of long-term care facilities, a medically vulnerable population at high risk for severe RSV disease, were less likely to be vaccinated. Furthermore, patients with Medicaid insurance and uninsured patients, those with a high school education only, and those residing in areas of high social vulnerability were also less likely to be vaccinated, highlighting socioeconomic differences in vaccine uptake that have been observed in studies of other vaccines and national data on RSV vaccination.^10,11,16,17,18,26,27,28^

Lack of awareness of RSV disease and vaccine recommendations as well as these sociodemographic differences may have contributed to low overall uptake of the vaccine. Almost half of the respondents had not heard of RSV; among unvaccinated participants, approximately 80% were either unsure whether they were eligible or stated they were ineligible for RSV vaccination. Knowledge of RSV disease and vaccine eligibility was lower among participants with less educational attainment and those residing in communities with high social vulnerability. Notably, 41.7% of unvaccinated patients reported being open to future RSV vaccination; they represent a “movable middle” group that is undecided about vaccination, with most indicating that a conversation with a health care professional about the vaccine might facilitate their receipt of vaccine. Recommendations by health care professionals are known to be the most effective strategy to increase vaccine uptake.^29^ Findings from this analysis suggest that focused outreach about RSV disease risk and vaccination recommendations to adults eligible for but undecided about RSV vaccination could increase RSV vaccination uptake in subsequent seasons.

Despite intent to vaccinate, multiple barriers to vaccine access may have also contributed to low RSV vaccine uptake in the first season. First, the SCDM recommendation, by definition, does not include a default to vaccinate everyone in a particular age or risk group; therefore, SCDM recommendations can be perceived by health care professionals as a limited call to action and confusing and time-consuming to implement.^6,9,10,30^ Additionally, while 74.1% of patients in this analysis received the RSV vaccine at pharmacies or grocery stores (consistent with national survey findings^10^), most of the unvaccinated patients who were open to future RSV vaccination cited wanting to speak with their physician before vaccine receipt. However, because RSV vaccines are covered under Medicare Part D (pharmacy benefit),^31^ many clinics may not stock these vaccines due to complex reimbursement paperwork. This mismatch between a desire for recommendation by a health care professional and limited ability to receive RSV vaccination in clinical settings at the time of a recommendation because of Medicare Part D coverage suggests logistical barriers to vaccinate.

In adjusted analyses, there were signals of socioeconomic barriers to RSV vaccination. Patients with at least some college or technical training and those living in communities with low or moderate social vulnerability were more likely to receive RSV vaccination. By contrast, patients with Medicaid insurance and uninsured patients, surrogates for socioeconomic vulnerability, were less likely to be vaccinated. Additionally, receipt of COVID-19 and influenza vaccines was associated with receipt of RSV vaccine. Prior studies have documented an inverse association between elements of social vulnerability, such as lower educational level and community SVI, and uptake of COVID-19 and influenza vaccines.^16,17,18^ Together, these findings suggest that there are shared bases in differences in respiratory virus vaccination uptake. Efforts to address sociodemographic differences in RSV vaccination may be most effective if they include a holistic approach that addresses general barriers to access to preventive care and overall factors associated with vaccine hesitancy. For communities with increased social vulnerability, offering vaccine information and vaccination in a wide range of community-based locations such as emergency departments, mobile clinics in high SVI areas, and long-term care facilities could increase vaccine uptake.^32^

### Limitations

Several limitations should be considered when interpreting these findings. First, this analysis included hospitalized adults who may not represent the general population of older adults but is likely to represent some of the most medically vulnerable older adults who may benefit most from RSV vaccination. Second, only 54.8% of patients completed knowledge and attitude questions about RSV disease and vaccines; respondents were more likely to be younger, to speak English, and to have less severe illness. Although this analysis provides important signals of sociodemographic differences in RSV vaccination during the first season of use, additional efforts are needed to understand factors influencing RSV vaccine acceptance and receipt in different populations. Third, although the analysis examined insurance status in a largely insured population, Medicare Part D coverage status was not collected specifically. Fourth, this analysis was not designed to estimate RSV vaccination coverage, and the proportion of patients who received RSV vaccine should not be interpreted as an estimate of national RSV vaccine coverage. The proportion of RSV-vaccinated patients in this cross-sectional analysis was lower than national coverage estimates, which represent cumulative coverage throughout the season.

## Conclusions

In this cross-sectional study of hospitalized adults, knowledge of RSV disease and RSV vaccine eligibility was low. Among unvaccinated patients during the 2023-2024 season, 41.7% reported being open to future RSV vaccination, indicating the opportunity to expand RSV disease prevention substantially. The updated RSV recommendations for all adults 75 years and older and those aged 60 to 74 years at increased risk of RSV disease simplify the previous SCDM recommendation.^6^ Ongoing monitoring in future RSV seasons is needed to determine whether simplified recommendations, increases in awareness of RSV disease and vaccine information, and expansion in access to primary prevention services will lead to improved RSV vaccination coverage among older adults at higher risk for severe disease.

## Notes

Supplement 1. **eAppendix.** Investigators and Collaborators **eMethods.** Eligibility, Enrollment, Vaccination Status, and Human Participants Research Determination **eTable 1.** Underlying Medical Conditions Obtained Through Medical Record Review **eTable 2.** Characteristics of Patients With and Without Interview Data **eFigure 1.** Self-Reported Locations of RSV Vaccination Among Adults Hospitalized With RSV-Negative Acute Respiratory Illness **eFigure 2.** Self-Reported RSV Vaccination Status and Attitudes Related to Receipt and Nonreceipt of RSV Vaccine

Supplement 2.
Data Sharing Statement
