# Supplementary material for: Patient- and Community-Level Characteristics Associated With Respiratory Syncytial Virus Vaccination
Source: JAMA Netw Open. 2025 Apr 1;8(4):e252841. doi: 10.1001/jamanetworkopen.2025.2841 (PMC11962666; doi:10.1001/jamanetworkopen.2025.2841)
Supplement: Supplement 1. — eAppendix. Investigators and Collaborators eMethods. Eligibility, Enrollment, Vaccination Status, and Human Participants Research Determination eTable 1. Underlying Medical Conditions Obtained Through Medical Record Review eTable 2. Characteristics of Patients With and Without Interview Data eFigure 1. Self-Reported Locations of RSV Vaccination Among Adults Hospitalized With RSV-Negative Acute Respiratory Illness eFigure 2. Self-Reported RSV Vaccination Status and Attitudes Related to Receipt and Nonreceipt of RSV Vaccine [file jamanetwopen-e252841-s001.pdf]

Supplementary Online Content

Surie D, Yuengling KA, Safdar B, et al. Patient- and community-level characteristics associated with respiratory syncytial virus vaccination. *JAMA Netw Open.* 2025;8(4):e252841. doi:10.1001/jamanetworkopen.2025.2841

eAppendix. Investigators and Collaborators

eMethods. Eligibility, Enrollment, Vaccination Status, and Human Participants Research Determination

eTable 1. Underlying Medical Conditions Obtained Through Medical Record Review

eTable 2. Characteristics of Patients With and Without Interview Data

eFigure 1. Self-Reported Locations of RSV Vaccination Among Adults Hospitalized With RSV-Negative Acute Respiratory Illness

eFigure 2. Self-Reported RSV Vaccination Status and Attitudes Related to Receipt and Nonreceipt of RSV Vaccine

This supplementary material has been provided by the authors to give readers additional information about their work.

© 2025 Surie D et al. *JAMA Network Open*.

eAppendix

IVY

INVESTIGATING RESPIRATORY VIRUSES IN THE ACUTELY ILL

Investigators and Collaborators in the **I**nvestigating respiratory **V**iruses in the Acutel**Y** Ill (**IVY**) Network

- Baylor, Scott and White, Temple, Texas: Manju Gaglani, Shekhar Ghamande, Tresa McNeal
- Baylor Scott and White Health, Dallas, Texas: Manju Gaglani, Cristie Columbus, Robert L. Gottlieb, Catherine Raver, Ashley Bychkowsky, Symone Dunkley, Tammy Fisher, Daniela Gonzalez, Therissa Grefsrud, Mariana Hurutado-Rodriguez, Gabriela Perez
- Baystate Medical Center, Springfield, Massachusetts: Jay S. Steingrub
- Beth Israel Medical Center, Boston Massachusetts: Nathan I. Shapiro
- Centers for Disease Control and Prevention (CDC), Atlanta, Georgia: Diya Surie, Fatimah S. Dawood, Katharine A. Yuengling, Nathaniel M. Lewis, Sascha Ellington, Rachel E. Rutkowski
- Cleveland Clinic, Cleveland, Ohio: Abhijit Duggal
- Emory University, Atlanta, Georgia: Laurence W. Busse, Jamie Felzer, William S. Bender
- Hennepin County Medical Center, Minneapolis, Minnesota: Matthew E. Prekker, Anne Frosch, Heidi Erickson, Audrey Hendrickson, Mary O'Rourke, Samuel Boes, Kowsar Hurreh, Julianna Prohofsky, Danielle Laibly, Joel Dumonsau
- Intermountain Medical Center, Murray, Utah: Ithan D. Peltan, Samuel M. Brown, Jenna Lumpkin, Shandi Poulson, Joslyn Bassett, Vineela Thumma
- Johns Hopkins University, Baltimore, Maryland: David N. Hager, Safa Saeed
- Montefiore Medical Center, Bronx, New York: Michelle N. Gong, Amira Mohamed
- The Ohio State Medical Center, Columbus, Ohio: Matthew Exline, Sarah Karow, Maryiam Khan, Conner Lang, Brooke Lee, Rushil Madan, Amanie Rasul, Connor Snyder, Rasha Alrifae, Zachary Lewald, Manisha Pathak, Reece Wilson, Gabrielle Swoope
- Oregon Health and Sciences University, Portland, Oregon: Akram Khan, Catherine L. Hough, Riya Mathew, Amelie Voutilainen, Pavel Savinskiy, Adrian Hernandez-Frausto, Edvinas Pocius, Emily Tribbett, Genesis Briceno, Jose Pena, Sarah Pellicer
- Stanford University, Stanford, California: Jennifer G. Wilson, Samantha Ferguson, Lily Lau, Vanessa Pitre, Grace Kyin-Ye Tam, Cynthia Perez, Leonard Basobas, Alexandra June Gordon
- University of Arizona: Jarrod Mosier, Karen Lutrick, Cameron Hypes, Beth Salvagio Campbell
- University of California-Los Angeles, Los Angeles, California: Nida Qadir, Steven Y. Chang, Cody Tran, Sukantha Chandrasekaran, Omai Garner
- University of Colorado, Aurora, Colorado: Adit A. Ginde, Samantha Simon, Amanda Martinez, Amy Sullivan, Laura Aguilar-Marquez, Erika Alor, Yvette Evans, Jacob Rademacher
- University of Iowa, Iowa City, Iowa: Nicholas M. Mohr, Anne Zepeski, Paul Nassar, Jacob Hampton, Cathy Fairfield, Heath Gibbs, Courtney Feitsam
- University of Miami, Miami, Florida: Christopher Mallow, Carolina Rivas
- University of Michigan, Ann Arbor, Michigan: Adam S. Lauring, Emily T. Martin, Aleda M. Leis, Weronika Valvano, Arnold S. Monto
- University of Utah, Salt Lake City, Utah: Estelle S. Harris, Riley McBride, Misty Yamane

© 2025 Surie D et al. *JAMA Network Open*.

- University of Washington, Seattle, Washington: Nicholas J. Johnson, Vasisht Srinivasan, Joshua Acidera, Katherine Elkort, Sarah Stucky, Amol Gajendragadkar, Maile McKeown, Leenay Coughlin
- Vanderbilt University Medical Center, Nashville, Tennessee: Wesley H. Self, Yuwei Zhu, H. Keipp Talbot, Carlos G. Grijalva, Natasha Halasa, James D. Chappell, Kelsey N. Womack, Jillian P. Rhoads, Adrienne Baughman, Sydney A. Swan, Cassandra A. Johnson, Todd W. Rice, Jonathan D. Casey, Paul W. Blair, Colleen Ratcliff, Ian D. Jones, Jakea Johnson, Laura L. Short, Lauren J. Ezzell, Margaret E. Whitsett, Rendie E. McHenry, Samarian J. Hargrave, Jennifer L. Luther, Claudia Guevara Pulido, Bryan P. M. Peterson, Shanice L. Cummings, Emma Claire Gauthier, Anna C. Jackson, Neekar S. Rashid
- Wake Forest University, Winston-Salem, North Carolina: Kevin W. Gibbs
- Washington University, St. Louis, Missouri: Jennie H. Kwon, Bijal Parikh, David McDonald, Carleigh Samuels, Lucy Vogt, Caroline O'Neil, Alyssa Valencia, Francesca Yerbic, Olivia Arter, Kim Vu
- Henry Ford Health, Detroit, Michigan: Ivana A. Vaughn, Mayur Ramesh, Lois E Lamerato, Khaled Almawri, Ishraaq Atkins, Jaleesa Clark, Alycia Lilla, Catherine McKeon, Mariia Numi, Shruti Tirumala, Katrina Williams Jean Ashley Lava, Melissa Resk, Sindhuja Koneru, Rachna Jayaprakash, Zina Pinderi
- Yale University, New Haven, Connecticut: Basmah Safdar, Anirudh Goyal, Ivan Velasquez, Uchechi Okoronkwo, Danielle Paquette

© 2025 Surie D et al. *JAMA Network Open*.

eMethods. Eligibility, Enrollment, Vaccination Status, and Human Participants Research Determination

1. Eligibility Criteria and Enrollment Practices

Patients were enrolled according to the eligibility criteria listed below. Enrollment teams attempted to enroll all patients with laboratory-confirmed RSV, SARS-CoV-2, and influenza based on clinical viral testing conducted at the enrolling hospital, if they met syndromic criteria for acute respiratory illness (ARI, see inclusion criteria below). For each enrolled patient with confirmed RSV, SARS-CoV-2 and influenza, enrollment teams also enrolled, within two weeks, at least one patient who met syndromic criteria for ARI and tested negative for RSV, SARS-CoV-2, or influenza based on clinical viral testing.

In addition to clinical viral testing in the local hospital, nasal swabs were collected from enrolled patients and systematically tested at a central laboratory (Vanderbilt University Medical Center) for RSV, SARS-CoV-2, and influenza by reverse transcription-polymerase chain reaction (RT-PCR) using standardized methods. Patients with negative clinical and central test results for RSV were included in this analysis. If patients tested positive for SARS-CoV-2 or influenza by either clinical or central testing, they were also included in this analysis. This is because patients who test negative for RSV are considered classic "test-negative" controls that represent the source population for vaccine uptake in test-negative design vaccine effectiveness studies, which is the enrollment framework used by the IVY Network.

Enrollment Inclusion Criteria:

1. Age ≥18 years old.
2. Hospital admission or in an emergency department awaiting hospital admission.
3. Symptoms and/or signs compatible with an acute respiratory illness, including at least 1 of the following: fever; cough; shortness of breath; hypoxemia (for patients not on chronic supplemental oxygen, hypoxemia is defined as: SpO2 <92% or use of supplemental oxygen to maintain SpO2 ≥92%; for patients on chronic supplemental oxygen, hypoxemia is defined as SpO2 below the patient's baseline SpO2 or an escalation of supplemental oxygen use to maintain the baseline SpO2 value); new pulmonary findings on chest imaging consistent with pneumonia.
4. Clinically obtained test that is positive (for RSV cases) or negative (for test-negative control-patients) for acute RSV after onset of symptoms for the current illness. The test may be obtained before or after hospital arrival. Examples of acute viral tests include RT-PCR tests, nucleic acid amplification tests (NAAT), and antigen tests. Serology testing may not be used for eligibility.

Enrollment Exclusion Criteria:

1. Patient was admitted to the hospital more than 7 days ago (based on this exclusion criterion, patients must be enrolled within 7 days of hospital admission).
2. The first RSV test is known to have occurred more than 10 days after onset of acute viral infection symptoms/signs listed in inclusion criterion #3. Patients with unknown symptoms/signs onset date may be enrolled.
3. First test for acute RSV is more than 3 days after hospital admission.
4. Previously enrolled in this surveillance program within the prior 30 days.
5. For test-negative control-patients, any positive test for acute RSV, SARS-CoV-2 or influenza virus infection after symptom onset for the current illness.
6. For test-negative control-patients, inability to obtain an upper respiratory sample for central laboratory testing within 10 days of symptom onset for the current illness.

© 2025 Surie D et al. *JAMA Network Open*.

2. Classification of RSV Vaccination Status
Vaccination status for RSV was determined from electronic medical records (EMR), state or jurisdictional registries, and by self- (or proxy-) report. Available vaccination data from each of these sources were collected, including date and location of vaccine administration, vaccine manufacturer, and lot number. Final vaccination status was determined by combining data from verified documented sources (EMR and registry data) as well as plausible self- (or proxy-) report based on date and location of RSV vaccination.

During the period of this analysis, the only two licensed and recommended RSV vaccine products were: 1) Arexvy (GlaxoSmithKline) and 2) Abrysvo (Pfizer, Inc.). Patients who received either of these vaccines before hospitalization, regardless of the timing of vaccination to hospitalization, were classified as "vaccinated" in this analysis.

3. Human Subjects Research Determination

3.1 Public Health Surveillance

This project was conducted as non-research public health surveillance in accordance with the Code of Federal Regulations (CRF) and guidance from the Office for Human Research Protections (OHRP).

During the conduct of this project, we collected information and biospecimens requested by CDC to monitor respiratory virus epidemiology and vaccine effectiveness. This project was supported and requested by CDC, which serves as the public health authority for this project. Activities were limited to those necessary for CDC to monitor respiratory viruses for the purposes of public health.

The criteria for non-research public health surveillance activities are outlined in 45 CFR 46.102(*l*)(2):

> "Public health surveillance activities, including the collection and testing of information or biospecimens, conducted, supported, requested, ordered, required, or authorized by a public health authority. Such activities are limited to those necessary to allow a public health authority to identify, monitor, assess, or investigate potential public health signals, onsets of disease outbreaks, or conditions of public health importance (including trends, signals, risk factors, patterns in diseases, or increases in injuries from using consumer products). Such activities include those associated with providing timely situational awareness and priority setting during the course of an event or crisis that threatens public health (including natural or man-made disasters)."

OHRP provides the following three criteria for an activity to be considered public health surveillance. :

- The activity must be a public health surveillance activity (45 CFR 46.102(*l*)(2));
- The activity must be conducted, supported, requested, ordered, required, or authorized by a public health authority (45 CFR 46.102(k) and 46.102(*l*)(2)); and
- The activity must be limited to that necessary to allow a public health authority to identify, monitor, assess, or investigate potential public health signals, onsets of disease outbreaks, or conditions of public health importance (including trends, signals, risk factors, patterns in diseases, or increases in injuries from using consumer products) (45 CFR 46.102(*l*)(2)).

© 2025 Surie D et al. *JAMA Network Open*.

eTable 1. Underlying Medical Conditions Obtained Through Medical Record Review

Condition categories included for this analysis are as follows: cardiovascular disease, pulmonary disease, neurologic disease, endocrine disease, kidney disease, gastrointestinal disease, hematologic disease, and immunocompromising conditions.

**Cardiovascular disease**
Heart failure
Peripheral vascular disease that limits mobility
Prior myocardial infarction
Cardiac arrhythmias including atrial fibrillation and ventricular arrhythmias
Valvular heart disease
Hypertension
**Pulmonary disease**
Asthma
Chronic obstructive pulmonary disease
Cystic fibrosis
Pulmonary fibrosis
Pulmonary hypertension
Home oxygen use (except at night for sleep disorder)
Tracheostomy
Home non-invasive ventilation use (except at night for sleep disorder)
Home invasive ventilation use
**Neurologic disease**
Dementia
Prior stroke
Prior transient ischemic attack (TIA, “mini-stroke”)
Brain or spinal cord injury with loss of limb function
Cerebral palsy
Muscular dystrophy
Multiple sclerosis
Myasthenia gravis
Anterolateral sclerosis (ALS)
**Endocrine disease**
Diabetes mellitus without end organ damage
Diabetes mellitus with end organ damage
Adrenal insufficiency
Hypothyroidism
**Kidney disease**
Chronic kidney disease without chronic kidney replacement therapy
End stage renal disease on chronic kidney replacement therapy (including hemodialysis or peritoneal dialysis)
**Gastrointestinal disease**
Feeding through a tube
Inflammatory bowel disease including Crohn's Disease or Ulcerative Colitis
Cirrhosis (clinical diagnosis of cirrhosis)
Chronic liver disease without cirrhosis
Peptic ulcer disease
**Hematologic disease**

© 2025 Surie D et al. *JAMA Network Open*.

Sickle cell disease (all variants)
Coagulopathy or other bleeding disorder, such as hemophilia
Chronic anemia
Thalassemia
**Immunocompromising conditions**
Active solid tumor or hematologic malignancy (defined as newly diagnosed malignancy or malignancy treatment within the past 6 months)
Solid organ transplant
Hematopoietic cell transplant (HCT)
HIV infection
Primary immunodeficiency
Use of immunosuppressive medication in the past 30 days
Other conditions that cause moderate or severe immunosuppression

© 2025 Surie D et al. *JAMA Network Open*.

eTable 2. Characteristics of Patients With and Without Interview Data

Characteristic	Patients **with** interview data<br><br>N= 3,694<br><br>n (%)	Patients **without** interview data<br><br>N= 3,052<br><br>n (%)	*P* value
Age in years, median (IQR)	71 (65–78)	75 (68–83)	<.001
Age group, years			
60–74	2346 (63.5)	1492 (48.9)	<.001
≥75	1348 (36.5)	1560 (51.1)	
Female sex	1892 (51.2)	1559 (51.1)	.91
Race and ethnicity[a]			
Black, non-Hispanic	805 (22.1)	483 (16.4)	<.001
Hispanic or Latino, any race	266 (7.3)	433 (14.7)	
White, non-Hispanic	2443 (67.0)	1856 (62.8)	
Other race, other ethnicity	130 (3.6)	183 (6.2)	
Non-English speaking	141 (3.8)	452 (14.8)	<.001
Hypoxemic at admission[b]	2137 (57.9)	1809 (59.3)	.24
Admitted to the ICU[c]	161 (12.2)	268 (23.2)	<.001
Dementia	85 (2.3)	339 (11.1)	<.001

Abbreviations: IQR = interquartile range; ICU = intensive care unit

[a] Race and ethnicity were self-reported and categorized as Black, non-Hispanic; Hispanic or Latino, any race; White, non-Hispanic; Other race, non-Hispanic (includes Asian, Native American or Alaska Native, and native Hawaiian or other Pacific Islander); and Other (includes patients who self-reported their race and ethnicity as “Other” and those for whom race and ethnicity were unknown).

[b] Hypoxemia was defined as having at least one of the following within 24 hours of hospital arrival: an SpO2 <92% or supplemental oxygen use for patients without chronic supplemental oxygen use, or escalation of respiratory support for patients who do receive chronic supplemental oxygen

[c] Data on ICU admission were available for 1319 patients with interview data and 1153 patients without interview data

© 2025 Surie D et al. *JAMA Network Open*.

eFigure 1. Self-Reported Locations of RSV Vaccination* Among Adults Hospitalized with RSV-Negative Acute Respiratory Illness — IVY Network, 26 Hospitals, 20 US States, October 1, 2023–April 30, 2024 (N=6,746)

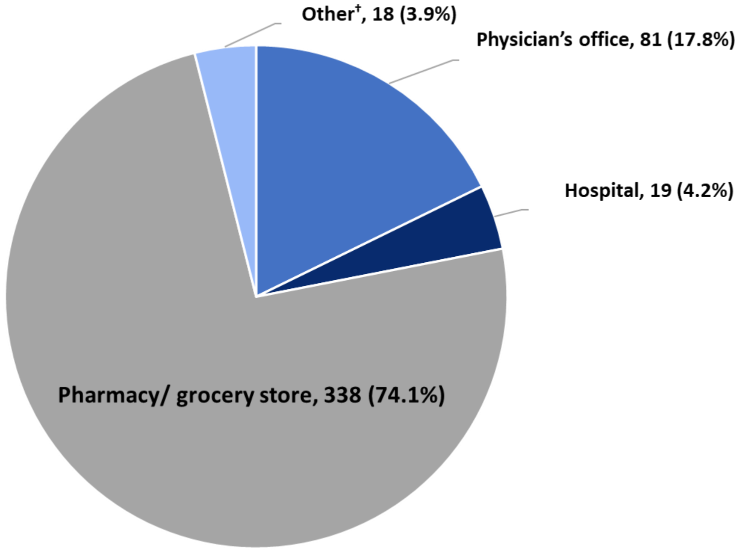

*Of 700 RSV vaccinated patients, 456 (65.1%) had reported location of RSV vaccination
†Other locations included the following: senior or community center (n=7), long-term care facility (n=6), state or local health department (n=1), home health service (n=1), dialysis center (n=1), research facility (n=1), and workplace (n=1)

© 2025 Surie D et al. *JAMA Network Open*.

eFigure 2. Self- Self-Reported RSV Vaccination Status and Attitudes Related to Receipt and Nonreceipt of RSV Vaccine — IVY Network, 26 hospitals, 20 US States, October 1, 2023–April 30, 2024 (N=3,694*)

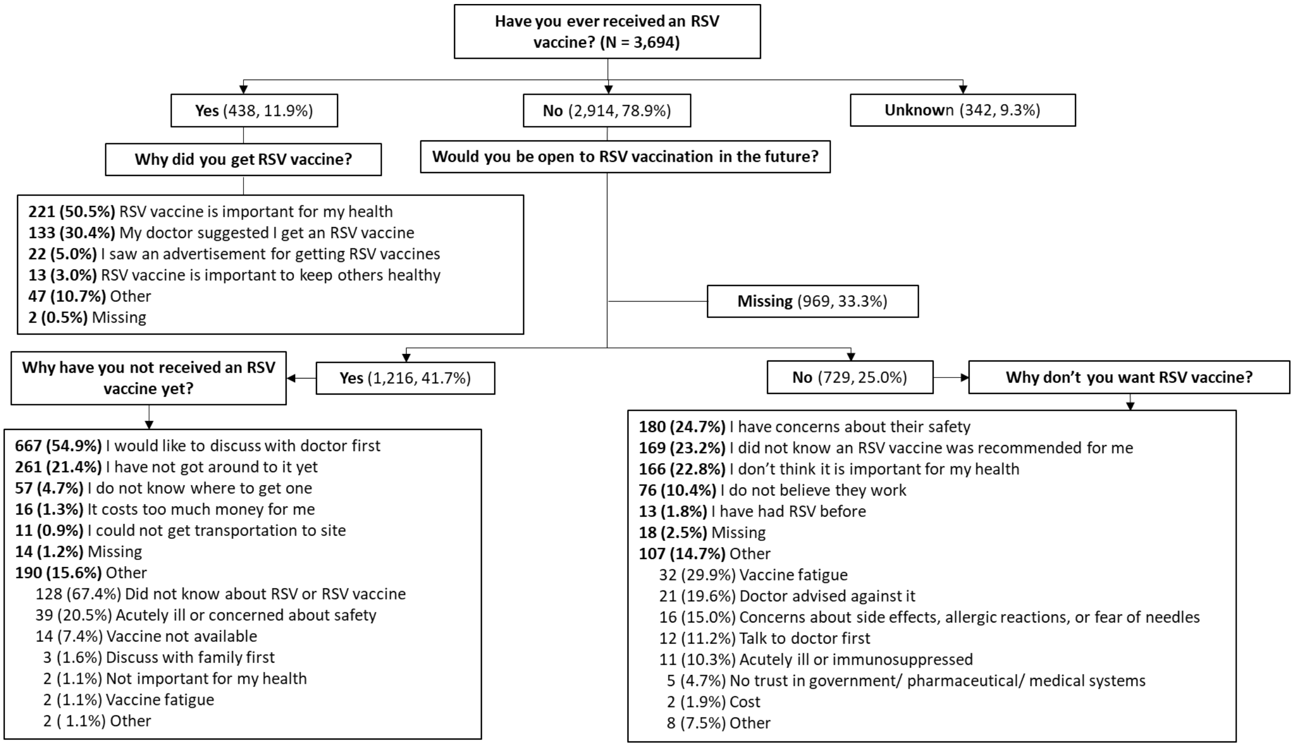

* Of, 6746 patients included in the analysis population, a total of 3,052 (45.2%) patients were excluded from these responses due to at least one of the following reasons: not completing a patient interview, not completing the self-reported vaccination history component of the interview or having mixed patient and proxy responses to these questions.

© 2025 Surie D et al. *JAMA Network Open*.
