# Supplementary material for: Patient- and Community-Level Characteristics Associated With Respiratory Syncytial Virus Vaccination
Source: JAMA Netw Open. 2025 Apr 1;8(4):e252841. doi: 10.1001/jamanetworkopen.2025.2841 (PMC11962666; doi:10.1001/jamanetworkopen.2025.2841)
Supplement: Supplement 2. — Data Sharing Statement [file jamanetwopen-e252841-s002.pdf]

Data Sharing Statement

Surie. Patient- and Community-Level Characteristics Associated With Respiratory Syncytial Virus Vaccination. *JAMA Netw Open*. Published April 01, 2025.
doi:10.1001/jamanetworkopen.2025.2841

Data
Data available: No
